# 

**Published:** 1977

**Authors:** 


JULY/OCTOBER 1977

VOLUME 92 (iii/iv)

NUMBER 343/344

Published Quarterly

Annual Subscription £4 Post Free

### BRISTOL MEDICO-CHIRURGICAL JOURNAL

### Contents

3 **Who Cares for Children ?**
S. D. M. Court

9 **Forty Years of Obstetrics in General Practice**
Christopher Morgans

Established 1883

### BRISTOL MEDICO-CHIRURGICAL JOURNAL

Incorporating the Medical Journal of the South West

### Editorial Committee

EDITOR
*Dr. D. S. Reeves*
*Southmead Hospital, Bristol, BS10 5NB*
ASSISTANT EDITOR
*Dr. A. P. Radford*
MEMBERS
*Dr. Rhys Davies*
*Dr. M. B. Lennard*
*Dr. D. W. Wright*
*The Hon. Treasurer of the Society*
*The Hon. Secretary of the Society*
SECRETARY
*Mr. B. P. Jones, The Library, Medical School,*
*University Walk, Bristol, BS8 1TD*

### Bristol Medico-Chirurgical Society

PRESIDENT 1977/78
*Dr. A. T. M. Roberts*
PRESIDENT ELECT
*H. K. Lucas, Esq.*
HONORARY SECRETARY
*A. J. Webb, Esq., 7 Percival Road, Bristol, BS8 3LE*
HONORARY TREASURER
*Dr. J. Penry, Southmead Hospital, Bristol, BS10 5NB*

The Society was founded in 1874. In 1883 publication of the Journal began and has continued quarterly ever since then. During the years 1949 to 1962 it appeared under the title of 'The Medical Journal of the South West'.

### Notice to Contributors

*The Journal is especially concerned to provide a place for the recording of medical thought, interests, and practice in and around Bristol. It therefore welcomes articles that, as well as being of immediate interest to members, will document the local and contemporary medical scene for our successors.*

*Original articles are invited provided that they have not been published elsewhere. Illustrations should be supplied ready for reproduction. Line drawings should be in Indian ink on firm white paper or board. The author will be informed if it is found necessary to ask him to pay for the printing of photographs etc.*

*The following contributions will be especially welcome; notes of forthcoming events, meetings held, degrees conferred, staff appointments, honours and public appointments and other local news.*

*Advice on preparation can be obtained from the Editor.*

Printed by the University of Bristol Printing Unit

